# Adherence to Consolidated Standards of Reporting Trials (CONSORT) Guidelines for Reporting Safety Outcomes in Trials of Cannabinoids for Chronic Pain: Protocol for a Systematic Review

**DOI:** 10.2196/11637

**Published:** 2019-01-28

**Authors:** Mohammed M Mohiuddin, Glenio Mizubuti, Simon Haroutounian, Shannon Smith, Fiona Campbell, Rex Park, Ian Gilron

**Affiliations:** 1 Department of Anesthesiology & Perioperative Medicine Queen's University Kingston, ON Canada; 2 Washington University Pain Center Washington University School of Medicine St. Louis, MO United States; 3 University of Rochester Medical Center School of Medicine and Dentistry University of Rochester Rochester, NY United States; 4 Department of Anesthesia and Pain Medicine University of Toronto Toronto, ON Canada; 5 Department of Biomedical and Molecular Sciences Queen's University Kingston, ON Canada; 6 Centre for Neuroscience Studies Queen's University Kingston, ON Canada

**Keywords:** adverse events, adverse event reporting, cannabis, cannabinoids, cannabidiol, chronic pain, clinical trial, systematic review, marijuana, safety, tetrahydrocannabinol

## Abstract

**Background:**

Chronic pain affects a significant proportion of the population and presents a major challenge to clinicians and pain specialists. Despite the availability of pharmacologic treatment options such as opioids, many patients continue to experience persistent pain. Cannabinoids present an alternative option with some data on efficacy; however, to date, a systematic review of adverse events (AEs) assessment and reporting in randomized clinical trials (RCTs) involving cannabinoids has not been performed. As a result, it is unclear whether a clear profile of cannabinoid-associated AEs has been accurately detailed in the literature. As cannabinoids are likely to become readily available for patients in the near future, it is important to study how well AEs have been reported in trials so that the safety profile of cannabinoids can be better understood.

**Objective:**

With a potentially enormous shift toward cannabinoid use for managing chronic pain and spasticity, this study aims to reveal the adequacy of AE reporting and cannabinoid-specific AEs in this setting. Spasticity is a major contributor to chronic pain in patients with multiple sclerosis (MS), with a comorbidity of 75%. Many cannabinoid studies have been performed in MS-related painful spasticity with relevant pain outcomes, and these studies will be included in this review for comprehensiveness. The primary outcome will be the quality of AE assessment and reporting by adherence to the Consolidated Standards of Reporting Trials (CONSORT) guidelines. Secondary outcomes will include the type of AE, method of AE reporting, severity of AE, frequency of AEs, patient withdrawals, and reasons for withdrawals.

**Methods:**

We will perform a systematic review by searching for primary reports of double-blind, randomized controlled trials of cannabinoids compared with placebo and any active comparator treatments for chronic pain, with a primary outcome directly related to pain (eg, pain intensity, pain relief, and pain-related interference). We will search the following databases: MEDLINE, Embase, Cochrane Library, and PsycINFO. RevMan software will be used for meta-analysis.

**Results:**

The protocol has been registered on the International Prospective Register of Systematic Reviews (CRD42018100401). The project was funded in 2018 and screening has been completed. Data extraction is under way and the first results are expected to be submitted for publication in January or February 2019.

**Conclusions:**

This review will better elucidate the safety of cannabinoids for the treatment of chronic pain and spasticity through identifying gaps in the literature for AE reporting. Like in any new therapy, it is essential that accurate information surrounding the safety and efficacy of cannabinoids be clearly outlined and identified to balance the benefit and harm described for patients.

**Trial Registration:**

PROSPERO CRD42018100401; https://www.crd.york.ac.uk/prospero/display_record.php?RecordID=100401

**International Registered Report Identifier (IRRID):**

DERR1-10.2196/11637

## Introduction

Chronic pain affects a significant proportion of the population and presents a major challenge to clinicians and pain specialists. Chronic pain can be defined as ongoing pain lasting longer than 3-6 months and negatively affecting a patient’s well-being [1]. Market research suggests that up to 1.5 billion people globally suffer from some form of chronic pain [2]. A World Health Organization study of developed countries conducted in 1998 found chronic pain was reported in 22% of patients coming in for primary care [3]. Recent studies of chronic pain have found prevalence rates ranging from 18% to 50% [4-6]. Interference in activities due to chronic pain included self-care, work, sexual life, and sleep quality. With limited access to evidence-based physical and psychological chronic pain interventions [7,8], medications remain the mainstay of treatment. The socioeconomic burden of chronic pain is also substantial, costing just the US economy alone anywhere from US $560 to US $635 billion per year when direct and indirect costs are factored in [3]. Numerous classes of therapeutic agents have been studied and used to treat chronic pain, such as opioids, anticonvulsants, and antidepressants [3]. Despite the availability of these treatment options, chronic pain persists in patients, perhaps because of a lack of analgesic efficacy or poor tolerability due to adverse effects [9,10]. Additionally, patients with chronic pain report poorer health and increased symptoms of anxiety and depression [11,12], which can be disabling and impede their ability to carry out a functional, productive lifestyle. While opioids are currently used to treat various forms of chronic pain, they are sometimes associated with significant risks, including opioid addictions and opioid-related deaths [13], that make them an unfavorable form of treatment.

Cannabis has been used for centuries for several indications including the treatment of pain. Following research on cannabinoids and their mechanism of action in the late 20th century [14], the endocannabinoid system (ECS) was discovered. The ECS, composed of endogenous cannabinoids, their associated enzymes, and cannabinoid receptors [15], plays a significant role in regulating neuropathic pain through neuromodulation and immunomodulation [14]. Currently, there are three main categories of cannabinoids identified in the literature: phytocannabinoids from the cannabis plant, synthetic cannabinoids, and ECSs [14]. *Cannabis sativa* contains over 400 different chemical compounds, 61 of which are cannabinoids acting on the ECS [16]. There are two main cannabinoid receptors that have been identified in the central and peripheral nervous system: the G-protein-coupled cannabinoid receptor type 1 and type 2 (Table 1). Delta 9-tetrahydrocannabinol and cannabidiol are two cannabinoids found in the resin [17] of the cannabis plant that interact with cannabinoid receptor type 1 and cannabinoid receptor type 2 (Table 2) [18].

With the legalization and subsequent increased availability of cannabis emerging in various parts of the world, it is expected that more individuals will seek cannabinoids as a means of relieving various forms of pain [19], including chronic neuropathic pain. Indeed, some research has shown that the wider use of cannabinoids has resulted in a decrease of opioid-related deaths [20]. It is important to note that there are very little data describing the dose-response effect of various cannabinoids to treat chronic pain. However, studies of cannabis smoking in humans suggest a therapeutic window for analgesia, whereby going above the window may increase pain and adverse effects such as nausea, drowsiness, vomiting, headaches, and memory impairment [21-23]. Thus, it is vital for health care and policy decisions that adverse events (AEs) associated with cannabis use for chronic pain management are clearly outlined and identified to balance the benefit and harm described for patients. The Consolidated Standards of Reporting Trials (CONSORT) guidelines [24], with a harms extension [25], were established to improve the AE assessment and reporting in clinical trials. Recent systematic reviews have studied the adherence of randomized clinical trials (RCTs) to CONSORT guidelines [26-28]. Studies of analgesic RCTs in 3 major pain journals (European Journal of Pain, Journal of Pain, and PAIN) found that approximately 4 out of the 10 recommendations of the CONSORT guidelines were not being met [26-28]. This is of significant clinical concern, as it suggests there is a gap in our understanding of the safety of treatments for chronic pain. If a similar gap in safety assessment and reporting exists in trials for cannabinoids treating chronic pain, then it is vital that clinicians and patients alike are made aware of the harms. This may also promote an improvement in the methodology of AE collection and reporting in clinical trials so that there is an emphasis on safety data along with efficacy.

To date, a systematic review of AE assessment and reporting in RCTs involving cannabinoids for chronic pain has not been performed. The Preferred Reporting Items for Systematic Reviews and Meta-Analyses harms checklist [29] was established to improve harms reporting in systematic reviews.

**Table 1:** The cannabinoid receptors of the endocannabinoid system and their mechanism of action.

Cannabinoid receptor	Expression in the body	Mechanism of action
CB_1_ R^a^	Presynaptic terminals of peripheral nociceptors and neurons in the dorsal root ganglion and spinal cord [12,13].	Presynaptic activation of CB_1_ R results in the reduction of nociceptive transmission by reducing a release of neurotransmitters such as gamma-aminobutyric acid and glutamate [13].
CB_2_ R^b^	Cells such as astrocytes, microglia, and macrophages along with lymphoid tissues in the spleen and lungs.	Activation of CB_2_ R decreases the release of proinflammatory cytokines such as interleukins, interferon gamma, and tumor necrosis factor alpha, ultimately resulting in reduced inflammation, nociception, and hyperalgesia.

^a^CB_1_ R: cannabinoid receptor type 1.

^b^CB_2_ R: cannabinoid receptor type 2.

**Table 2:** The active metabolites of cannabis and their interactions with the endocannabinoid system.

Active compound	Interacting receptor	Mechanism of action
Delta 9-THC^a^	CB_1_ R^b^ and CB_2_ R^c^	Delta 9-THC acts as a partial agonist on CB_1_ R and CB_2_ R, resulting in effects such as reduction in pain, increased appetite, and changes in emotional and cognitive processes [13].
CBD^d^	CB_1_ R, 5-HT1A^e^ serotonergic and TYPV1-2^f^ vanilloid receptors	CBD acts as a negative allosteric modulator of CB_1_ R and numerous other receptors such as 5-HT1A serotonergic and TYPV1-2 vanilloid receptors. CBD has various pharmacological effects, such as being anxiolytic, anti-inflammatory, antiemetic, and antipsychotic. It has also been shown to have antioxidative, anti-inflammatory, and neuroprotective effects at lower doses [14].

^a^THC: tetrahydrocannabinol.

^b^CB_1_ R: cannabinoid receptor type 1.

^c^CB_2_ R: cannabinoid receptor type 2.

^d^CBD: cannabidiol.

^e^5HT1A: 5-hydroxytryptamine 1A.

^f^TYPV1-2: transient receptor potential vanilloid 1-2.

An analysis of the adherence of systematic reviews to the Preferred Reporting Items for Systematic Reviews and Meta-Analyses harms checklist has not been performed, and our review seeks to advance the goals of this checklist. Within the scope of patients experiencing chronic pain are those suffering from painful spasticity in multiple sclerosis (MS), which is known to be highly associated with pain, with comorbidity of 75% and treatment frequently using cannabinoids [30]. Many cannabinoid studies have been performed in MS-related painful spasticity with relevant pain outcomes, and these studies will be included in this review. With a potentially enormous shift toward cannabinoid use for managing chronic pain, it is essential that accurate information surrounding both the harms as well as the efficacy of cannabinoids be clearly outlined.

The use of medical cannabis is becoming increasingly legalized across developed nations, such as in Canada (2018), allowing for many members of the population to obtain this substance from dispensaries with ease. This legalization may also result in clinicians prescribing cannabis more frequently than before. It is critical that both the safety and efficacy of medications be well established before prescribing to patients. The findings of this project will help elucidate the AEs associated with cannabinoid use for treating chronic pain and, importantly, reveal the appropriateness and accuracy of AE reporting. This will help to form a clearer picture of the risk-benefit profile of cannabinoids. Furthermore, the findings from this project may help inform both clinicians and the public on the nature of cannabis in a clinical context, helping to ensure it is not used inappropriately. These results may also inform politicians and commissioning bodies who may make changes on relevant policies based on research findings. Ultimately, this project seeks to promote the safety of patients in whom cannabinoids reduce chronic pain. The primary outcome will be the quality of AE assessment and reporting by adherence to CONSORT guidelines. Secondary outcomes will include the type of AE, method of AE reporting, AE severity, frequency of AEs, patient withdrawals, and reasons for withdrawals.

## Methods

### Eligibility Criteria

We will perform a systematic review by searching for primary reports of double-blind, randomized controlled trials of cannabinoids compared with a placebo and any active comparator treatments studied for chronic noncancer pain, with a primary outcome directly related to pain (eg, pain intensity, pain relief, pain-related interference, other analgesic consumption, etc). Chronic pain has been defined as persistent or recurrent pain lasting longer than 3 months [31]. We will include RCTs with parallel and crossover design published in English peer-reviewed journals from all years until 2018. RCTs involving patients with any type of chronic noncancer pain and patients with spasticity in multiple sclerosis will be included, with no restrictions with regards to setting, age, or country. RCTs involving patients with terminal cancer or other terminal illness will be excluded. The intervention will be cannabinoid-based medicines, which include phytocannabinoids (herbal cannabis, hashish, and marijuana), plant-derived cannabinoids (nabiximols and delta 9-tetrahydrocannabivarin), synthetic cannabinoids (cannabinoid analogs, levonantradol, and nabilone), and synthetic drugs interacting with the ECS.

We will include drugs administered at any dose or by any route, such as smoking, vaporizing, transdermal applications, and ingesting, as well as RCTs comparing cannabinoids to any active comparator or placebo. Examples of active comparators include nonsteroidal anti-inflammatory drugs, antidepressant medications, anticonvulsant medications, and opioids.

### Information Sources and Search Strategy

We will search the following electronic bibliographic databases: MEDLINE, Embase, PsycINFO, and Cochrane Library (Cochrane Database of Systematic Reviews, Cochrane Central Register of Controlled Trials, and Cochrane Methodology Register). Additionally, RCTs registered on clinicaltrials.gov will also be searched. All publication years until 2018 will be included, and other gray literature sources will be excluded.

### Search Strategy

An experienced librarian from the Faculty of Health Sciences at Queen’s University will be involved in creating the search strategy and conducting the search. A sample strategy for MEDLINE is included here. We will apply the following Medical Subject Headings (MeSH) when retrieving articles: Cannabis, Cannabinoids, Cannabidiol, Marijuana Smoking, Tetrahydrocannabinol, and tetrahydrocannabinol-cannabidiol [25]. Any articles with the keywords, cannabis, cannabinoid, cannabidiol, marijuana, marihuana, dronabinol, tetrahydrocannabinol, or tetrahydrocannabinol will also be retrieved. From this set of articles, we will pass the Medical Subject Heading “Pain,” or title keyword “pain” through the “Clinical Queries: therapy/narrow” filter to render the final set of articles [32]. From this, the phrases “chronic pain,” “neuropathic pain,” “neuropathy,” and “painful neuropathy,” along with “pain” will be utilized to obtain the relevant literature.

The search strategy will include only terms relating to or describing the intervention. We will combine the terms with the Cochrane MEDLINE filter for controlled trials of interventions. Where available, the search terms will be adapted for use with other bibliographic databases in combination with database-specific filters for controlled trials.

We will only select studies published in English and those published since the inception of each bibliographic database. We will record the date the searches are run and will rerun them just before the final analyses to retrieve any further studies for inclusion.

### Data Extraction, Sorting, and Selection

The selection process will involve two independent reviewers on the research team who will work in pairs for screening, eligibility, and inclusion in the meta-analysis through each phase of the review, so that the work is duplicated. We will screen potential titles across all databases included in the search. The abstracts will then be assessed to ensure the correct treatment in the appropriate setting is being studied. The full report for each abstract will then be screened to ensure that the criteria of the review protocol are met. If there are any disagreements between reviewers, this will be resolved by consulting with a third reviewer from the team. Data that are missing or not reported in trials will be reported in the analysis. The data collection process will involve creating an electronic data extraction form to be used to obtain the relevant data forms. Each reviewer will perform this independently, so that the extraction is done in duplicate. We will resolve any discrepancies through discussion with the review authors and the principal investigator when necessary.

We will extract the following variables for each trial: year of publication, country, type of cannabinoid, design, pain condition, number of treatment groups, comparators, frequency of drug administration, study duration, appropriateness of masking, route of drug delivery, dose administered, primary outcome, number of participants randomized, number of study sites, study sponsor, and journal impact factor from the year in which the article was published.

As the CONSORT Extension for Harms was published in 2004, we will collect data from all published studies but do a comparison from pre-2004 articles to post-2004 in order to assess any difference in AE reporting after the published guidelines.

We will define AEs as all adverse treatment effects occurring during a trial, following the definition in the CONSORT Extension for Harms statement [25]. This definition will range from tolerability to safety issues. Studies will be evaluated for the quality of AE reporting by their adherence to the CONSORT Extension for Harms, each being coded using the descriptors. We will consider partial fulfillment of a recommendation as satisfactory in order to facilitate comparing trials. The studies will be analyzed for their assessment method, categorized as “spontaneous reporting from patient,” “asked an open-ended question by investigator to report any AEs,” “patient diary,” “specific AEs assessed by the patient,” “specific AEs assessed by direct questioning by an investigator,” “multiple methods of AE assessment,” or “not specified.” We will also review the timing of AE assessments in addition to the time frame in which the drug was administered. The specific time periods, and reporting of the severity of the AEs will also be recorded. Data will be extracted on study patient withdrawals, whether studies outline reasons for withdrawals, and numbers of withdrawals per study. We will also extract the specific reported AE outcomes from each study and outline the most frequently reported AEs in both the drug and placebo groups.

### Outcomes and Prioritization

The primary outcome will be the quality of AE assessment and reporting as evaluated by the adherence of included RCTs to each of the 10 CONSORT Extension for Harms recommendations. We selected this primary outcome as these are well-established guidelines for RCTs to follow with respect to safety assessment and reporting. Additionally, it allows for a standardized, quantitative assessment of studies and comparison across studies.

Secondary outcomes will include the type, method, reporting, severity, and frequency of the AE; the timing of AE reporting [33]; patient withdrawals; and reasons for patient withdrawals. We chose these secondary outcomes because they allow for a comprehensive assessment of the specific AEs experienced by patients in these trials and any potential biases in reporting.

### Risk of Bias in Individual Studies

In order to assess internal validity, the Cochrane risk of bias tool will be employed [34]. The risk of bias in the included studies will be independently assessed by two review authors by considering the following characteristics: (1) randomization sequence generation: was the allocation sequence adequately generated?; (2) treatment allocation concealment: was the allocated treatment adequately concealed from study participants and clinicians and other health care or research staff at the enrollment stage?; (3) blinding: were the personnel assessing outcomes and analyzing data sufficiently blinded to the intervention allocation throughout the trial?; (4) completeness of outcome data: were participant exclusions, attrition, and incomplete outcome data adequately addressed in the published report?; (5) selective outcome reporting: is there evidence of selective outcome reporting and might this have affected the study results?; and (6) other sources of bias: was the trial apparently free of any other problems that could produce a high risk of bias?

Disagreements between the review authors over the risk of bias in particular studies will be resolved by discussion, with the involvement of a third review author where necessary.

### Data Integration and Synthesis

A descriptive approach to data analysis and reporting will be used. Each item of the CONSORT recommendations will be assigned equal weight in the analysis. The percentage of trials fulfilling each CONSORT Extension for Harms recommendation, and the number of recommendations fulfilled by each trial will be recorded.

We will present adherence to the CONSORT Extension for Harms guidelines as an overall score out of 10. These imputation methods are acceptable, but we will record what method each study used to accommodate for missing data. Careful discussion with the principal investigator will be had to ensure accuracy in analysis for these cases. A narrative synthesis of the findings will be provided from the included studies, structured around the type of intervention, target population characteristics, type of outcome, and intervention content. The review will also present summaries of the intervention effects for each study by calculating risk ratios (for dichotomous outcomes) or standardized mean differences (for continuous outcomes). The heterogeneity of studies will also be assessed using the Higgins I^2^ [35]. For statistical analysis, this review will use RevMan software version 5 (Cochrane Collaboration). Finally, the quality of evidence will be assessed according to the GRADE system [36].

Where studies have used the same type of intervention and comparator with the same outcome measure, we will pool results using a random-effects meta-analysis, with standardized mean differences for continuous outcomes and risk ratios for binary outcomes, and calculate 95% CIs and 2-sided *P* values for each outcome. We will also assess evidence of publication bias.

## Results

We have received funding from the Queen’s University Anesthesiology Vandewater Studentship, allowing for the project to begin. The PROSPERO registration number is CRD42018100401. The project was funded in 2018 and screening has been completed. Data extraction is under way and the first results are expected to be submitted for publication in January or February 2019.

## Discussion

Currently, there is an opioid crisis affecting society, resulting in a reduction in health-related quality of life, increased rates of substance use disorder, and loss of life. Chronic pain is experienced by a significant proportion of the population and, currently, clinicians face challenges in creating a holistic and effective pain management plan for patients that does not revolve around opioids. With limited access to evidence-based physical and psychological chronic pain interventions, medications remain the mainstay of treatment. Current pharmaceutical agents, including opioids, are at best moderately effective in the treatment of chronic pain and are commonly associated with significant harms. The findings of this project can ultimately aid clinicians and the general population in making evidence-based decisions for their care, in the context of a chronic and sometimes debilitating condition.

## Appendix

Multimedia Appendix 1 Peer-reviewer report from Queen's University.

## Abbreviations

AE: adverse event
CONSORT: Consolidated Standards of Reporting Trials
ECS: endocannabinoid system
MS: multiple sclerosis
RCT: randomized clinical trial
