# Supplementary material for: Adherence to Consolidated Standards of Reporting Trials (CONSORT) Guidelines for Reporting Safety Outcomes in Trials of Cannabinoids for Chronic Pain: Protocol for a Systematic Review
Source: JMIR Res Protoc. 2019 Jan 28;8(1):e11637. doi: 10.2196/11637 (PMC6369421; doi:10.2196/11637)
Supplement: Multimedia Appendix 1 [file resprot_v8i1e11637_app1.pdf]

Dr. L. Wang (MD, FRCPC, MSc)
DEPARTMENT OF ANESTHESIOLOGY & PERIOPERATIVE MEDICINE
Queen's University, Faculty of Health Sciences,
Kingston, Ontario K7L 2V7

Kingston General Hospital
76 Stuart Street

Tel: 613 548-7827
Fax: 613 548-1375

June 1, 2018

Dr. I. Gilron
Department of Anesthesiology & Perioperative Medicine
Queen's University & Kingston General Hospital,
Kingston, Ontario K7L 2V7

Dear Dr. Gilron,

Your recent submission to the Department of Anesthesiology and Perioperative Medicine Vandewater Endowed Studentship for a summer student (Mohammed Mohiuddin) underwent a peer review process during which it was independently reviewed and scored by at least 2 members of the Department of Anesthesiology.

I am pleased to inform you that your application entitled **Assessment and Reporting of Safety Outcomes in Clinical Trials of Cannabinoids for Chronic Pain** was recommended for funding in the amount of $2,500 for which you have agreed to match an additional $2,500. Dana Thompson-Green can assist you with the set-up of your research account which is required before funds can be transferred. We will require a progress report (with financials) to be submitted upon project completion.

Congratulations on your successful application!

Best Regards,

Louie Wang, Research Director
Department of Anesthesiology &
Perioperative Medicine Research Committee

Overall average score= 9/10

The proposal is of high potential not only for publication but for laying the groundwork for future projects. This will be excellent experience for Mohammed and potentially help in laying the foundation for his future career as a clinician researcher.
